# Regulation of macrophage plasticity by circCCDC7^19-13^ through HSP90 inhibition suppresses prostate cancer progression and metastasis: a translational study

**DOI:** 10.1097/JS9.0000000000002895

**Published:** 2025-07-01

**Authors:** Bisheng Cheng, Jianghua Yang, Wenxue Huang, Tianlong Luo, Lingfeng Li, Jilin Wu, Qianghua Zhou, Ruilin Zhuang, Qiong Wang, Kewei Xu, Peng Wu, Hai Huang

**Affiliations:** a Department of Urology, Nanfang Hospital, Southern Medical University, Guangzhou, China; b Department of Urology, Sun Yat-sen Memorial Hospital, Sun Yat-sen University, Guangzhou, China; c Department of Urology, Peking University People’s Hospital, Beijing, China; d Department of Urology, Sun Yat-sen University Cancer Center, Guangzhou, China; e Southern Medical University, Guangzhou, China; f Guangdong Provincial Key Laboratory of Malignant Tumor Epigenetics and Gene Regulation, Sun Yat-Sen Memorial Hospital, Sun Yat-Sen University, Guangzhou, China; g Guangdong Provincial Clinical Research Center for Urological Diseases, Sun Yat-Sen Memorial Hospital, Sun Yat-Sen University, Guangzhou, China; h Department of Urology, The Sixth Affiliated Hospital of Guangzhou Medical University, Qingyuan, Guangdong, China

**Keywords:** ferroptosis, prostate cancer, tumor-associated macrophages, tumor microenvironment

## Abstract

**Background:**

Macrophages play a crucial role in cancer development by differentiating into M1 or M2 phenotypes with opposing functions. The conversion of M2 to M1 macrophages has significant implications in cancer treatment. Non-coding RNA, such as circRNA and lncRNA, have been shown to regulate macrophage polarization by specific pathways. However, the molecular mechanism of this conversion process is still not fully understood.

**Method:**

In this study, we investigated the role of circCCDC7_19-13_ in regulating macrophage polarization in prostate cancer (PCA). We examined the expression level of circCCDC7_19-13_ in tumor-associated macrophages (TAMs) and M2 macrophages in PCA. We used in vitro experiments to investigate the effects of transfection of circCCDC7_19-13_ overexpression Lentivirus on M2 polarization and the expression level of M2 cytokines. We also assessed the ability of TAM to promote malignant behavior in PCA cells. Furthermore, we explored the downstream target of circCCDC7_19-13_ and its role in macrophage polarization.

**Results:**

We found that circCCDC7_19-13_ was significantly lower in TAMs and M2 macrophages in PCA. Overexpression of circCCDC7_19-13_ inhibited M2 polarization and downregulated the expression level of M2 cytokines. Overexpression of circCCDC7_19-13_ also reduced the ability of TAM to promote malignant behavior in PCA cells. Our study demonstrated that HSP90 was a downstream target of circCCDC7_19-13_, which could regulate macrophage polarization based on inhibition of ferroptosis. Overexpression of HSP90 restored the phenotype of circCCDC7_19-13_ overexpressing macrophages. CircCCD7_19-13_ overexpression of TAMs inhibited the growth and metastasis of PC3M cells.

**Conclusions:**

Our findings suggest that circCCDC7_19-13_ may alter macrophage polarization by inducing ferroptosis and targeting HSP90, which can decrease the ratio of M2 phenotype TAMs and inhibit the progression and metastasis of PCA. The results of this study provide new insights for clarifying the molecular mechanism of macrophage polarization and have reference value for targeted treatment of PCA.

## Introduction

The tumor microenvironment (TME) plays an important role in regulating the physiological activities of tumors, and it is composed of complex components, mainly including immune cells, stromal cells and extracellular matrix (ECM). Extensive research has elucidated the profound influence of the tumor microenvironment (TME) on the pathological mechanisms and prognostic outcomes of various types of tumors^[1-3]^. Among the cells in the TME, macrophages are particularly interesting due to their ability to differentiate into different phenotypes and perform diverse functions based on the signals they receive from the TME^[4-8]^. Macrophages are highly adaptable and differentiate into two phenotypes, namely, the M1 and M2 phenotypes, after exposure to different stimuli. M1 macrophages can secrete proinflammatory cytokines and enhance the inhibitory effect of the immune system on tumors, while M2 macrophages release anti-inflammatory factors and promote tumor growth^[9-12]^. However, the functions of these cells in the TME are very complex, and their precise functions and mechanisms of action remain incompletely understood.

Noncoding RNAs (ncRNAs) are RNA molecules that do not encode proteins but regulate gene expression via different mechanisms. Emerging research highlights the pivotal significance of noncoding RNAs (ncRNAs) in the regulation of macrophage function within the dynamic TME^[13-15]^. For example, microRNAs (miRNAs) can play regulatory roles in the differentiation of macrophages, mainly by stimulating specific signalling pathways such as the NF-kB pathway. MiR-155 has a particular downregulation effect on the protein expression of SOCS1, thereby promoting M1 polarization. In contrast, miR-223 promotes M2 macrophage polarization by targeting IRF1, a transcription factor that promotes M1 macrophage polarization. Additionally, long noncoding RNAs (lncRNAs), such as lncRNA-H19 and lncRNA-NEAT1, can regulate macrophage polarization and function by interacting with chromatin-modifying enzymes and other proteins. For instance, lncRNA-H19 promotes M2 polarization, while lncRNA-NEAT1 inhibits M1 polarization^[16,17]^. Circular RNAs (circRNAs) are a prevalent type of noncoding RNA characterized by their ability to form closed loops through specific splicing mechanisms. This unique feature highlights the potential of circRNAs to influence macrophage function and status, making them promising targets for cancer treatment. One key advantage of circRNAs is their resistance to exonucleases and reduced susceptibility to interference from other factors. They can effectively modulate gene expression at the transcriptional level by acting as microRNA sponges, working in conjunction to exert regulatory effects. Additionally, circRNAs have the capacity to modulate alternative splicing events and encode novel peptides, further expanding their regulatory capabilities^[18-20]^. Significant research has yielded compelling evidence that highlights the substantial impact of circular RNAs (circRNAs) on regulating the tumor microenvironment (TME) in the context of cancer^[15,21-24]^, indicating their potential for use as targets for cancer therapy.

CircCCDC7 is a circular RNA that is generated from exon 3 of the coiled-coil domain containing 7 (CCDC7) gene. It forms a closed circular structure that includes 401 nucleotides^[25]^ and is abnormally expressed in tumor tissues. The results of relevant experimental studies indicate that circCCDC7 can regulate various signaling pathways, thereby affecting the pathological processes and prognosis of cancer^[26-29]^. However, the mechanism underlying its function in the TME of PCA is still unclear and requires further research. Our previous research identified a novel circular RNA, namely, circCCDC7_19-13_, that is formed by trans-splicing of the 19th and 13th exons of CCDC7 and has the potential to encode peptides. In our study, we examined alterations in the expression pattern of circCCDC7_19-13_ in tumor-associated macrophages (TAMs) in the context of prostate cancer. Additionally, we explored the impact of these changes on TAM polarization and elucidated the underlying mechanisms involved.

Our findings revealed a significant downregulation of circCCDC7_19-13_ expression in M2 phynotype TAMs associated with PCa. Notably, the overexpression of circCCDC7_19-13_ induced ferroptosis in TAMs, simultaneously inhibiting the epithelial-mesenchymal transition (EMT) process and impeding the migration of PCa cells. Subsequent investigations have provided additional validation of heat shock protein 90 (HSP90) as a downstream target of circCCDC7_19-13_. Furthermore, in vivo experimental results have further substantiated this observation. Remarkably, this study reveals a novel regulatory mechanism for macrophage plasticity, shedding light on the ability of circCCDC7_19-13_ to modulate macrophage polarization by targeting HSP90. These findings emphasize the crucial role of circCCDC7_19-13_ in the regulation of prostate cancer (PCA) within the tumor microenvironment, while also contributing to a deeper comprehension of the mechanisms underlying macrophage plasticity. This research is compliant with the TITAN Guidelines 2025^[30]^.

**HIGHLIGHTS**

- CircCCDC7_19-13_ Regulation: CircCCDC7_19-13_ is downregulated in tumor-associated macrophages (TAMs) in prostate cancer tissues, suggesting its crucial role in the tumor microenvironment.
- Macrophage Polarization: Overexpression of circCCDC7_19-13_ induces ferroptosis in TAMs, promoting their polarization to the M1 phenotype and reducing M2 macrophage-associated pro-tumorigenic cytokine secretion.
- HSP90 Inhibition: CircCCDC7_19-13_ targets and inhibits HSP90, a key regulator in TAM polarization and ferroptosis resistance, revealing a novel mechanism of action.
- Suppression of Prostate Cancer Progression: Targeting circCCDC7_19-13_ significantly inhibits prostate cancer cell invasion, migration, and metastasis in both in vitro and in vivo models.
- Therapeutic Potential: CircCCDC7_19-13_ represents a promising therapeutic target for modulating the immune microenvironment and combating prostate cancer progression and metastasis.

## Materials and methods

### Clinical samples and isolation of primary macrophages

A total of 32 patients with this type of PCA were included in this study, and samples of cancer tissues and adjacent tissues were collected during radical resection treatment. All selected patients had complete pathological data and were diagnosed with PCA according to postoperative pathological analysis. The research plan was approved by the Ethics Committee of our hospital, and all the research subjects provided informed consent. In this study, samples from the two groups were separated using the Ficoll-Hypaque centrifugation method. The suspensions were centrifuged at low speed at room temperature for 10 minutes, the supernatants were discarded, and the resulting precipitates were resuspended in complete culture medium (Gibco, USA). First, the cell density was determined with a cell counter, and then, the cell concentration was adjusted to a concentration of 1 × 10^7^ cells/mL with DMEM. Based on the relevant instructions, D14 magnetic beads (BioLegend, 480026, USA) were added to the suspensions and then incubated in a suitable environment. At the end of the incubation, the obtained mixtures were centrifuged in a magnetic separator for 5 minutes to collect bead-bound cells. The free cells were removed, and the bead-bound cells were thoroughly washed with DMEM. According to the instructions, CD14^+^ cells were removed from the magnetic beads, the obtained suspensions were centrifuged at room temperature for 10 minutes, and the supernatants were removed. The obtained precipitates were fully suspended in DMEM, and the suspensions were cultured in a culture dish. The cells were cultured in DMEM supplemented with 10% FBS (ExCellBio, FSP500, China) and two antibodies (Gibco, 15140122, United States). During the cultivation process, the medium was changed every 2-3 days, and the cell morphology was closely observed and the results were accurately recorded (Figure S3, available at, http://links.lww.com/JS9/E497).

To extract primary tissue macrophages (PMs) from clinical prostatic hyperplasia tissues, CD14 magnetic beads were used for sorting. The PMs were subsequently incubated in DMEM supplemented with 10% FBS and 1% penicillin‒streptomycin. The incubator that was used to culture the PMs had a saturated humidity, with a temperature of 37 °C and 5% CO_2_.

### Induction of macrophage polarization

For the generation of M0-type macrophages, RAW264.7 cells were seeded in 6-well plates at a density of 6 × 10^^5^ cells per well. THP-1 cells were stimulated with phorbol 12-myristate 13-acetate (PMA) at a concentration of 200 ng/mL (Solarbio, China).To obtain M1 macrophages, M0 macrophages were stimulated with interferon-gamma (IFN-γ) at a concentration of 20 ng/mL (MedChemExpress, USA) and lipopolysaccharide (LPS) at a concentration of 500 ng/mL (Beyotime, China) for 48 hours. To generate M2 macrophages, M0 macrophages were stimulated with interleukin-4 (IL-4) at a concentration of 20 ng/mL (Beyotime, China) and interleukin-13 (IL-13) at a concentration of 20 ng/mL (PeproTech, USA) for 48 hours.

### RNA isolation, qPCR, and western blotting

The methods employed for RNA isolation and quantitative polymerase chain reaction (qPCR) were previously described, as well as the techniques for western blotting^[31,32]^. To isolate total RNA from cells, TRIzol® reagent was utilized, followed by reverse transcription using the HiScript II one-step qRT kit. For qPCR analysis in this study, SYBR qPCR Master Mix from regular domestic manufacturers and the classic ABI quantitative studio DX system were primarily employed. The relative expression of target genes was determined using the 2^-ΔΔCt^ method. The specific primers used for qPCR can be found in Supplementary Digital Content, Table S1, available at http://links.lww.com/JS9/E498.

Regarding immunoblotting, cell samples were lysed with RIPA lysis buffer, and the protein concentrations in the samples were determined using the Pierce BCA protein detection kit. The proteins were separated by SDS-PAGE and then swiftly transferred to PVDF membranes obtained from the United States. The membranes were subsequently incubated overnight at 4 degrees Celsius with primary specific antibodies(see Supplementary Digital Content, Table S2, available at http://links.lww.com/JS9/E498),which were then removed the following day. After the incubation, secondary antibodies conjugated to HRP were applied and incubated at 25°C for 60 seconds. The protein bands were finally visualized using the enhanced chemiluminescence method, and the resulting observations were processed and analyzed using ImageJ software.

### Cell lines and cell culture

In this study, the human PC3M prostate cancer cell line and other cell line samples were purchased from the Cell-type Culture Bank of the Chinese Academy of Sciences. These cell lines were incubated in RPMI-1640 medium. The medium was supplemented with appropriate concentrations of FBS and penicillin‒streptomycin; the cell lines were cultured at 37 °C in 5% CO_2_.To induce macrophage differentiation in vitro, the following protocols were utilized: (1) Tumor-associated macrophages (TAMs): Primary macrophages (PMs) and PC3M cells were co-incubated in Corning plates with a pore size of 0.4 µm for a duration of 2 days. (2) M0 macrophages: RAW264.7 cells were allowed to adhere to 6-well plates to generate M0 macrophages. (3) M1 macrophages: M0 macrophages were polarized into the M1 phenotype by treating them with appropriate concentrations of 20 ng/mL interferon-gamma (IFN-γ) and 500 ng/mL lipopolysaccharide (LPS) for 2 days. (4) M2 macrophages: M0 macrophages were polarized into the M2 phenotype by treating them with 20 ng/mL interleukin-4 (IL-4) and 20 ng/mL interleukin-13 (IL-13) for 2 days.

### Flow cytometric analyses

To prepare macrophages for analysis, they were harvested from the 6-well plates and washed twice with PBS. Subsequently, the cells were fixed in 4% paraformaldehyde for 10 minutes. Following fixation, 0.1% Triton was added to permeabilize the cells, and they were incubated for a designated period of time. After thorough washing with PBS, the cells were incubated on ice for 30 minutes with antibodies specific to cluster of differentiation 86 (CD86; FITC, Elabscience, China) and CD163 (BV421, Elabscience, China). Following two additional washes with PBS, the cells were resuspended in 200 µL of PBS, and the polarization index was determined.

### Cell invasion assay

The 8um membrane was coated with hydrated Matrigel (BD Biosciences Co., Ltd.), and PC3M cells were seeded into the upper chamber at a density of 2 × 10^^3^ cells/well with 100 µL of serum-free medium. The lower chamber was supplemented with a suspension containing either PMs, TAMs, or TAMs overexpressing circCCDC7_19-13_ (2 × 10^^5^ cells/mL, supplemented with 10% FBS). After a 36-hour incubation period, the medium in the upper chamber was discarded, and the cells were fixed with 4% paraformaldehyde for 30 minutes, followed by two washes with PBS. Subsequently, the cells were stained with 0.1% crystal violet (Solarbio, C8470, China) for 30 minutes at room temperature and washed three times with PBS. Cells that did not migrate from the upper chamber were gently removed using cotton swabs. The chambers were air-dried for 10 minutes, and images of five randomly selected fields were captured using a microscope (CX33, Olympus Corporation). Finally, the number of cells that had crossed the membrane was counted.

### Wound healing assays

Cell migration (wound healing) assays were performed by seeding PC3M and DU145 cells (6 × 10^5^ cells/well) in a six-well plate. In this part of the study, macrophages from each group were carefully seeded in Transwell chambers. To observe the migration of each group, when the PC3M cells reached 90% confluence, a special pipette tip was used to generate a horizontal scratch in the monolayer. Wash buffer was used to wash the samples repeatedly, and then, the floating cells were completely removed. The samples were transferred to a high-precision electron microscope, and images were captured; this corresponded to a time point of 0 h. The samples in each group were sequentially incubated for 2 days after moving the Transwell chambers to 6-well plates. Then, the upper chambers were removed and placed under a high-precision microscope to capture images, and the results were saved for subsequent data analysis.

### ROS assay

To measure reactive oxygen species (ROS) levels, macrophages were collected according to the actual experimental requirements, and then, the acquired samples were incubated with the DCFH-DA-ROS probe (5 µM) in the dark at 37 °C for half an hour. Wash buffer was used to wash the samples repeatedly and to resuspend the cells. Finally, the mean fluorescence intensity was determined within 30 min by using a flow cytometer (CytoFLEX S, Beckman).

### Phen green SK assay

To prepare the Phen Green SK (GLPBIO, GC40243, USA) stock solution, 1 mg of the dye was dissolved in 1 mL of DMSO. According to the actual experimental requirements, the appropriate number of macrophages was collected from each group, the macrophages were washed with wash buffer, and then, the samples were placed in 10 µM Phen Green SK and incubated for half an hour. After these steps, the absorbance values were determined at 485 and 535 nm by flow cytometry (CytoFLEX S, Beckman). The change in the fluorescence intensity of each sample was calculated.

### C11 BODIPY assay

To prepare a stock solution of C11 BODIPY (GLPBIO, C11 BODIPY 581/591, USA), 1 mg of the dye was dissolved in 1 mL of DMSO to a final concentration of 1 mM. This stock solution was stored at −20°C in the dark. For the experiments, the C11 BODIPY stock solution was diluted with buffer to a final concentration of 1 µM. Macrophages (2 × 10^5^ cells/mL) were collected from each group, washed, and incubated with 2 µM C11 BODIPY at room temperature for 15–30 minutes in the dark. The mean fluorescence intensities were measured using a flow cytometer (CytoFLEX S, Beckman) within 30 minutes. The oxidized form of the dye has an excitation wavelength of 460–495, while the reduced form of the dye has an excitation wavelength of 565–581 and an emission wavelength of 585–591.

### ELISA-based quantification of secreted levels of TNF-α and TGF-β

ELISA was used to determine the secreted levels of TNF-α and TGF-β. A commercial ELISA Kit (MEIMIAN, MM-51283H2/MM-0091H2, China) was used according to the manufacturer’s instructions. Supernatants were collected and diluted 1:2. The diluted samples were added to wells that were coated with TNF-α and TGF-β antibodies and incubated at 37 °C for 0.5 h. The absorbance of the samples was measured at 450 nm, after which the expression levels of TNF-α and TGF-β in all the samples were further determined with the standard curve.

### Intracellular glutathione (GSH) detection assay

Cells (5 × 10^5^) were collected and washed twice with PBS to remove any extracellular GSH. Cell lysis buffer was used if necessary, and the resulting lysates were centrifuged at high speed for 10 minutes. A total of 50 µL of a cell lysate or GSH standard was added to a microcentrifuge tube, and then, 150 µL of a 10% TCA solution was added. The tubes were vortexed and incubated on ice for 10 minutes, followed by high-speed centrifugation for 10 minutes. Then, the appropriate amount of EDTA disodium salt was prepared by mixing with water to obtain the EDTA solution that was required for this study. A total of 50 µL of supernatant was added to a new microcentrifuge tube, and 200 µL of 0.4 M EDTA solution and 25 µL of a 10 mM DTNB solution were added. The tubes were vortexed and incubated at room temperature for 10 min. After the above steps, the absorbance was measured at 412 nm with a spectrophotometer. The previously prepared 100 µL mixture was then moved to a corresponding 96-well plate for final analysis. The GSH concentration was then calculated using the GSH standard curve (Abbkine, KTB1600-48T, China).

### Colorimetric assay for measurement of intracellular ferrous iron levels

Cells (2 × 10^6^) were collected and washed twice with PBS to remove any extracellular iron. The cells were lysed using cell lysis buffer if necessary. The cells were centrifuged at 12 000 × g for 10 min. Then, 200 µL of a cell lysate or iron standard solution was added to a microcentrifuge tube. To this tube, 800 µL of 10% TCA solution was added, and the sample was vortex for 30 s. Then, the sample was centrifuge at high speed for 10 min. The ferrozine solution was prepared by dissolving 5 mM ferrozine and 50 mM sodium acetate trihydrate in deionized water. The previously prepared 100 µL of supernatant was added to a clean, sterile centrifuge tube. Then, 900 µL of iron zinc solution was added to achieve the desired ratio. The samples were incubated at room temperature for 10 min. After sample incubation, the absorbance was measured at 562 nm. Finally, the iron standard curve was used to further determine the concentration of ferrous ions in the samples (Leagene, TC1016, China).

### Plasmids and transfection

In the present study, a lentiviral vector carrying circCCDC7_19-13_ (labeled with GFP), a lentiviral vector carrying HSP90 (labelled with RFP), and an empty vector control were synthesized by Beijing Tsingke Biotechnology Co., Ltd. TAMs were transfected with the lentiviral vectors (circCCDC7_19-13_ or HSP90) at an MOI of 50 and cultured in RPMI-1640 medium supplemented with 10% FBS. Three days later, the fluorescence intensity was examined under a microscope, and the TAM transfection efficiency was further determined. After the above steps, stably transfected cell lines were selected and treated with 5 µg/mL puromycin for 7 days, and cell growth was closely observed; when the cells reached 80% confluence, the transfection efficiency was determined using RT-qPCR or other methods. The HSP 90 levels in the TAM samples were reduced by using HSP 90 inhibitors that were purchased from regular domestic manufacturers. In this paper, circCCDC7_19-13_ and an overexpression vector were used to transfect TAMs with HSP 90. Cell samples were collected 1 day later and stored for use in subsequent studies.

### In vivo popliteal LN metastasis and tumorigenesis assays

In this part of the study, healthy male BALB/c nude mice were used for the assessment of both popliteal lymph node metastasis and tumorigenesis. Laboratory animals were purchased from the Laboratory Animal Center of University and fed in a sterile barrier facility. These methods were previously described in the same studies mentioned above^[33,34]^. In brief, each control and PC3M cell sample was injected with mixed macrophages at a ratio of 1:4 into the footpad to establish the popliteal lymph node metastasis model. Subsequently, after the primary footpad tumor and popliteal lymph nodes were completely removed, they were embedded in paraffin for subsequent testing. The work has been reported in line with the ARRIVE criteria^[35]^.

### H&E *staining and immunohistochemistry*

The methodology used for immunohistochemistry was adapted from that described in our previous study^[36]^. Formalin-fixed prostate cancer (PCa) tissue sections were prepared as follows: first, the sections were deparaffinized using xylene and rehydrated through a series of diluted alcohols. Antigen retrieval was performed by microwaving the sections in 10 mM sodium citrate buffer (pH 6.0) for 10 minutes. To block endogenous peroxidase activity, the sections were treated with 0.3% H_2_O_2_ for 15 minutes, followed by incubation with goat serum to prevent nonspecific binding. The sections were then incubated overnight at 4°C with the primary antibodies of interest (see Supplementary Digital Content, Table S2, available at http://links.lww.com/JS9/E498). Following primary antibody incubation, the sections were washed three times with phosphate-buffered saline (PBS) to remove any unbound antibodies. Subsequently, the sections were incubated with appropriate secondary antibodies for 1 hour at room temperature. Visualization of the antibody-antigen complexes was achieved using the DAKO EnVision Detection System (Dako). Finally, the sections were counterstained with hematoxylin to visualize cell nuclei.

## Statistical analysis

All statistical analyses were performed using GraphPad Prism 9.0 software (GraphPad Software, USA). Data were presented as mean ± standard deviation (SD) unless otherwise specified. For comparisons between two groups, an unpaired Student’s t-test (two-tailed) was used if the data followed a normal distribution, while the Mann–Whitney U test was employed for non-normally distributed data. For comparisons among multiple groups, one-way or two-way analysis of variance (ANOVA) followed by Tukey’s post hoc test was conducted to evaluate statistical significance. For categorical data, the chi-square test or Fisher’s exact test was used where appropriate. Correlation analyses were conducted using Pearson’s correlation coefficient or Spearman’s rank correlation coefficient, depending on data distribution. Survival curves were generated using the Kaplan–Meier method, and differences between groups were assessed using the log-rank test. A *P*-value < 0.05 was considered statistically significant. Adjustments for multiple testing were performed using the Bonferroni correction where applicable. All experiments were performed with at least three biological replicates, and statistical methods were detailed in the respective figure legends.

## Results

### Downregulation of circCCDC7_19-13_ in macrophages from primary prostate cancer and bone metastases

**Figure 1.**
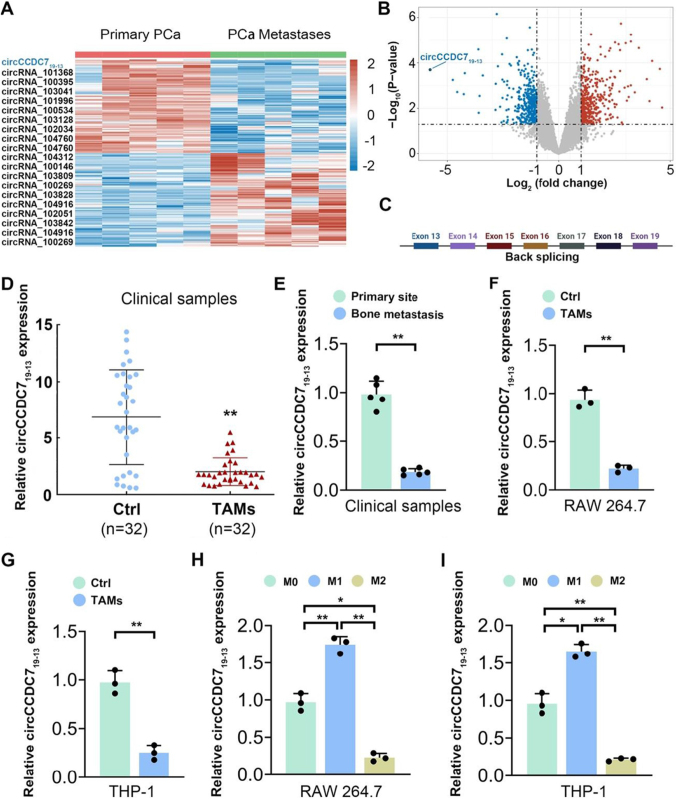
Differential expression of circCCDC7_19-13_ in macrophages from primary PCa and bone metastases. (A) Heatmap illustrating the expression profiles of circRNAs in macrophages sorted from primary PCa and PCa bone metastases using flow cytometry, followed by transcriptome sequencing. (B) Volcano plot showing the log_2_(fold change) versus -log_10_(*P*-value) for circRNAs in macrophages from primary PCa compared to PCa bone metastases. (C) Schematic of the circCCDC7_19-13_ genomic locus on chromosome 10 (chr 10 p11.22), showing back-splicing of exons 13 to 19. (D) Relative expression levels of circCCDC7_19-13_ in clinical samples of adjacent normal tissues (Ctrl, *n* = 32) and tumor-associated macrophages (TAMs, *n* = 32). (E) Relative expression levels of circCCDC7 in macrophages from clinical samples of primary PCa (*n* = 5) and bone metastases (*n* = 5). (F) Relative expression levels of circCCDC7_19-13_ in RAW 264.7 macrophages under control conditions (Ctrl) and after induction of TAMs. (G) Relative expression levels of circCCDC7_19-13_ in RAW THP-1 macrophages under control conditions (Ctrl) and after induction of TAMs. (H) Relative expression levels of circCCDC7_19-13_ in THP-1 macrophages under different polarization states: M0 (unpolarized), M1 (classically activated), and M2 (alternatively activated). (I) Relative expression levels of circCCDC7_19-13_ in RAW 264.7 macrophages under different polarization states. Data are presented as mean ± SD. *P < 0.05, **P < 0.01.

To explore the differential expression of circRNAs in macrophages associated with primary prostate cancer and PCa bone metastases, macrophages were sorted from these tissues using flow cytometry and subjected to transcriptome sequencing. Using log2|fold change| ≥ 2 and FDR < 0.05 as the selection criteria, we identified the target circRNAs. The heatmap revealed 10 up-regulated circRNAs and 10 significantly down-regulated circRNAs in macrophages, with circCCDC7_19-13_ being the most down-regulated in macrophages from bone metastases (Fig. 1A-B). First, the nucleotide sequence of circCCDC7_19-13_ was verified using circBase and circPrimer, and its head-to-tail splicing was confirmed through Sanger sequencing in our previous study^[37]^ (Fig. 1C).

To further validate the expression level of circCCDC7_19-13_ in TAMs, we employed a direct magnetic cell labeling method to isolate TAMs and macrophages from prostate cancer tissue samples and adjacent tissue samples obtained from 32 patients. Subsequently, we utilized an RT-qPCR detection method to quantify the levels of circCCDC7_19-13_ in these isolated cells. The results demonstrated a noticeable decrease in circCCDC7_19-13_ levels in TAMs derived from prostate cancer tissue samples compared to those derived from control adjacent tissue samples (Fig. 1D). Furthermore, our findings also revealed a significant reduction in circCCDC7_19-13_ expression levels in TAMs derived from metastatic lesions compared to macrophages derived from primary sites (Fig. 1E). Our preliminary research findings indicate that circCCDC7_19-13_ expression is down-regulated in tumor-associated macrophages (TAMs) within the prostate cancer tumor microenvironment (TME), suggesting its potential involvement in prostate cancer progression.

To investigate whether the reduced expression of circCCDC7_19-13_ in TAMs is influenced by prostate cancer cell stimulation, a transwell co-culture system was employed to simulate the TME. The expression levels of circCCDC7_19-13_ in cells RAW264.7 (co-cultured with TRAMP-C2 cells for 48 hours) and THP-1 cells (co-cultured with PC3M cells for 48 hours) were assessed using RT-qPCR. The results demonstrated that circCCDC7_19-13_ expression in both RAW264.7 and THP-1 cells was downregulated to varying extents following 48 hours of co-culture with prostate cancer cells (Fig. 1F,G). These findings suggest that the interaction between prostate cancer cells and TAMs may contribute to the decreased expression of circCCDC7_19-13_ in TAMs, highlighting its potential involvement in the prostate cancer microenvironment.

Previous related research has provided evidence indicating that tumor-associated macrophages (TAMs) share similarities with M2-like macrophages within the tumor microenvironment (TME)^[38]^. The present study thus induced RAW264.7 macrophages to differentiate into M1-type macrophages by stimulation with LPS/IFN-γ and into M2-type macrophages by stimulation with IL-4/IL-13, and then detected the changes in circCCDC7_19-13_ expression during M1/M2 polarization. Our findings revealed that the expression of circCCDC7_19-13_ was downregulated in M2 macrophages, while it was upregulated in M1-polarized macrophages (Fig. 1H). Consistent with these findings, circCCDC7_19-13_ expression was also downregulated in M2-polarized THP-1 cells (Fig. 1I). These experimental results provide evidence that circCCDC7_19-13_ expression is downregulated to varying degrees in both TAMs and M2-polarized macrophages, which is likely to be associated with the stimulation of tumor cells. The findings suggest that circCCDC7_19-13_ plays a role in TAM polarization, highlighting its potential impact on the tumor microenvironment.

**Figure 2.**
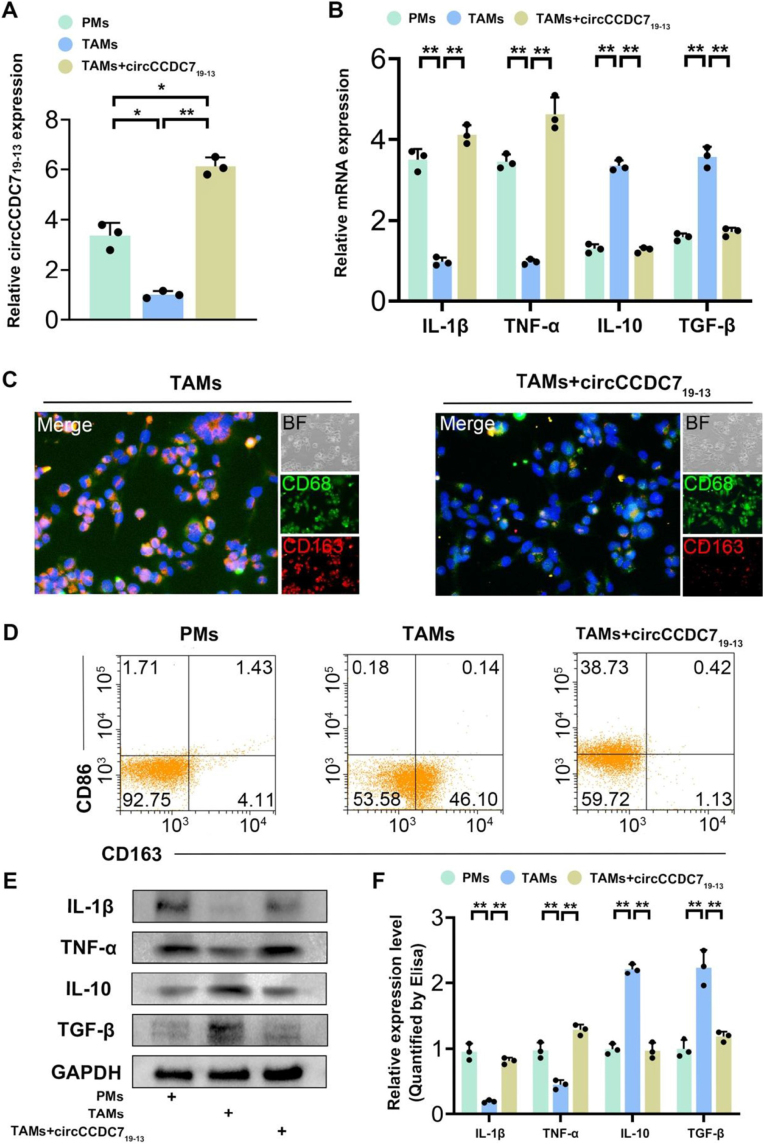
circCCDC7_19-13_ affects the polarization of TAMs and the secretion of cytokines. (A) RT-qPCR was employed to determine the expression levels of circCCDC7_19-13_ in cells transfected with circCCDC7_19-13_-overexpressing lentivirus. (B) The mRNA levels of M1-type secretory factors (INF-α and IL-1β) and M2-type secretory factors (TGF-β and IL-10) in PMs, TAMs, and TAMs circCCDC7_19-13_-overexpressing were detected using RT-qPCR. (C) An immunofluorescence assay was utilized to investigate CD86 and CD163 expression in TAMs and IAMs transfected with circCCDC7_19-13_-overexpressing lentivirus. (D) Flow cytometry was used to examine CD86 and CD163 protein levels in PMs, TAMs, and TAMs transfected with circCCDC7_19-13_-overexpressing lentivirus. (E, F) The protein levels of M1-type secretory factors (TNF-α and IL-1β) and M2-type secretory factors (TGF-β and IL-10) in PMs, TAMs, and TAMs transfected with circCCDC719-13-overexpressing lentivirus were analyzed using Western blot and ELISA. Data are presented as the mean ± SD of three independent experiments. *P* < 0.05 and *P < 0.01.

Primary macrophages (PMs) were collected and co-cultured with PC3M cells for 48 hours. Subsequently, a lentivirus overexpressing circCCDC7_19-13_ was utilized to transfect TAMs to ascertain the influence of circCCDC7_19-13_ on TAM plasticity (Fig. 2A). Immunofluorescence staining and flow cytometry were employed to assess the protein levels of CD86 and CD163, serving as markers for M1 and M2 type macrophages, respectively (Fig. 2C,D). The results demonstrated that TAMs exhibited lower CD86 levels and higher CD163 levels compared to PMs with the M0 phenotype. Interestingly, a substantial increase in circCCDC7_19-13_ levels led to a direct elevation in CD86 expression, whereas a decrease in CD163 expression further induced a phenotypic transition in TAMs from the M2 to the M1 phenotype.

Numerous studies have demonstrated that TAMs release significant amounts of cytokines that contribute to cancer progression^[39,40]^. We further investigates whether circCCDC7_19-13_ can modulate the secretion of tumor-related cytokines by TAMs. Using RT-qPCR and immunoblotting, we analyzed the mRNA and protein levels of M1- and M2-associated cytokines (Fig. 2B,E). Additionally, ELISA assays were performed to quantify cytokine secretion (Fig. 2F). The results showed that TAMs overexpressing circCCDC7_19-13_ secreted higher levels of M1 cytokines and lower levels of M2 cytokines. These findings indicate that elevated circCCDC7_19-13_ expression in TAMs alters both the polarization status and cytokine secretion capacity of these cells.

**Figure 3.**
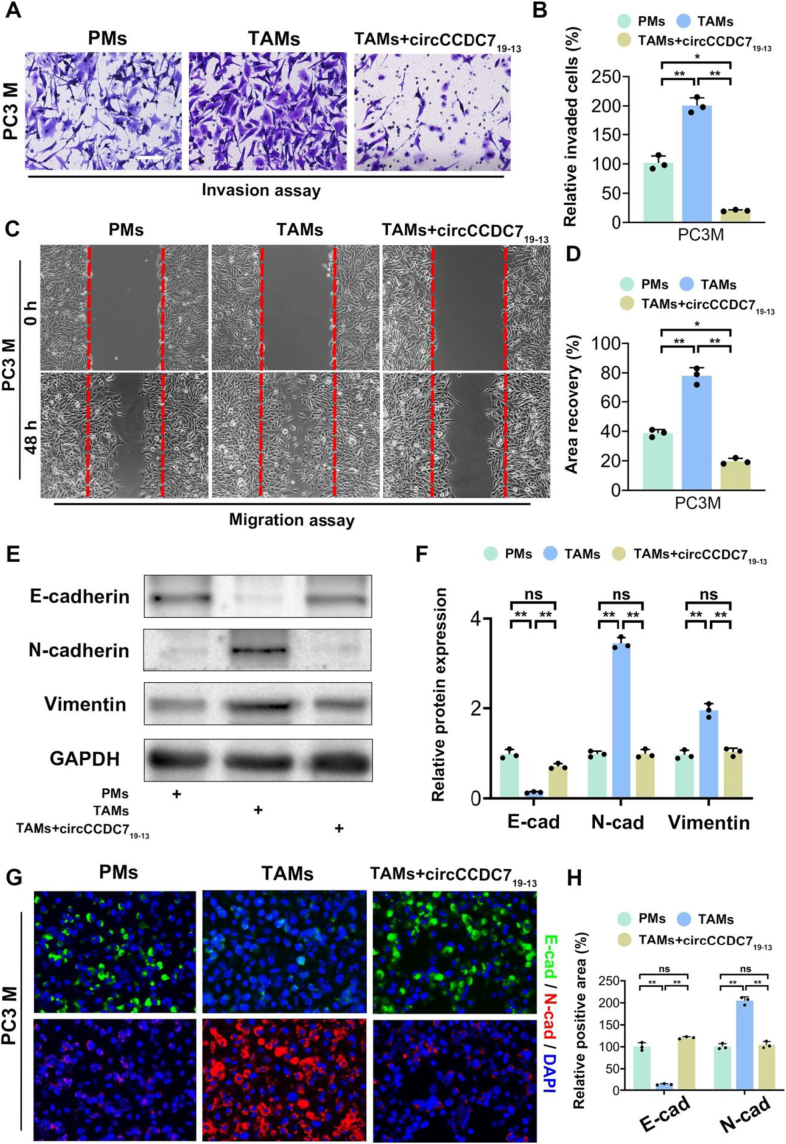
circCCDC7_19-13_ modulates TAM-mediated tumor invasion, migration, and EMT in PCa. (A) Representative images from Transwell invasion assays using PC3M cells, co-cultured with PMs, TAMs, or circCCDC7_19-13_-overexpressing TAMs (×200 magnification). (B) Quantification of invaded cells. (C) Representative images from wound healing assays at 0 and 48 hours (×100 magnification). (D) Quantification of area recovery. (E) Western blot analysis of E-cadherin (E-CA), N-cadherin (N-CA), and vimentin expression in PC3M cells. GAPDH was used as a loading control. (F) Quantification of relative protein expression. (G) Immunofluorescence staining for E-cadherin (green) and N-cadherin (red) in PC3M cells. (H) Quantification of immunofluorescence-positive areas. Data are presented as mean ± SD (*n* = 3); **p < 0.01.

Proliferation, invasion, and metastasis constitute the primary malignant behaviors of tumor cells. Nevertheless, TAMs in the tumor microenvironment (TME) predominantly facilitate tumor progression^[41]^. Epithelial mesenchymal transition (EMT) is known to promote tumor metastasis^[41]^, and TAMs can enhance the effects of EMT and exacerbate disease progression^[42-44]^. Multiple studies have demonstrated that M2-type macrophages secrete cytokines such as IL-10 and TGF-β, which contribute to the progression and metastasis of carcinoma^[45,46]^. In this study, following the verification of circCCDC7_19-13_’s influence on cytokine secretion by TAMs, the impact of circCCDC7_19-13_-overexpressing TAMs on the invasion and migration of PC3M prostate cancer cells was assessed. The findings demonstrated that PC3M cells, when co-cultured with TAMs, exhibited enhanced invasion and migration capabilities in comparison to the control group. However, these capabilities were mitigated when co-cultured with TAMs overexpressing circCCDC7_19-13_ (Fig. 3A-D).

Based on our study, we hypothesize that circCCDC7_19-13_ within TAMs may play a role in modulating epithelial-mesenchymaltransition (EMT) in prostate cancer cells. Further western blotting analysis revealed that the co-culture of PC3M cells with tumor-associated macrophages (TAMs) led to a significant decrease in E-cadherin levels, while concurrently showing varying degrees of increase in N-cadherin and vimentin levels. Conversely, PC3M cells co-cultured with circCCDC7_19-13_-overexpressing TAMs exhibited elevated E-cadherin and reduced N-cadherin and vimentin levels (Fig. 3E-H). These findings indicate that TAMs promote the invasion, migration, and EMT of PC3M cells, whereas circCCDC7_19-13_ can counteract these effects in TAMs. Thus, modulating circCCDC7_19-13_ may inhibit the vital process of prostate cancer.

**Figure 4.**
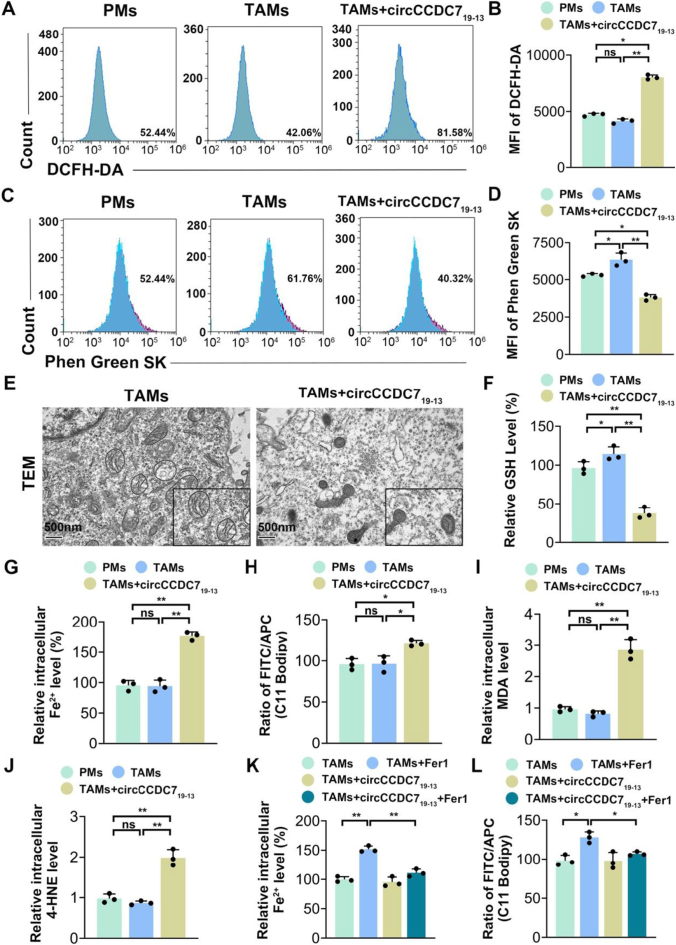
circCCDC7_19-13_ overexpression induces ferroptosis in TAMs. (A, B) ROS levels were measured using DCFH-DA staining by flow cytometry. (C, D) Intracellular Fe^2+^ levels were detected with Phen Green SK dye. (E) TEM images showing mitochondrial changes, including cristae loss and membrane density increases, in TAMs with and without circCCDC7_19-13_ overexpression. (F) GSH levels were determined by colorimetric assay. (G) Intracellular Fe^2+^ levels were quantified across groups. (H) Lipid peroxidation was assessed using the C11 BODIPY probe, expressed as the FITC/APC ratio. (I) MDA levels were measured as an indicator of oxidative damage. (J-L) Effects of Ferrostatin-1 (Fer-1) on Fe^2+^ levels and lipid peroxidation in TAMs overexpressing circCCDC7_19-13_. Data are expressed as mean ± SD from three independent experiments. *p < 0.05, **p < 0.01

Our previous research demonstrated that circCCDC7_19-13_ regulates the pathological process of ferroptosis in prostate cancer (PCa) cells and macrophages (Figure S1A-D, available at http://links.lww.com/JS9/E497). Ferroptosis, characterized by mitochondrial swelling, endoplasmic reticulum rupture, and lipid peroxidation, influences the recruitment, polarization, and activation of TAMs (tumor-associated macrophages)^[47]^. To further investigate how circCCDC7_19-13_ affects macrophage polarization, we assessed lipid peroxidation, intracellular Fe^2+^, and reactive oxygen species (ROS) levels using flow cytometry. Our results revealed that TAMs exhibited lower levels of lipid peroxidation, Fe^2+^, and ROS than PMs (primary macrophages). However, these markers were significantly increased in TAMs overexpressing circCCDC7_19-13_ (Fig. 4A-D). Using Transmission Electron Microscopy (TEM), we found that TAMs with high circCCDC7_19-13_ expression displayed distinct ferroptosis-associated changes, including reduced mitochondrial volume, increased bilayer membrane density, and loss of mitochondrial cristae (Fig. 4E).

Since glutathione (GSH) plays a vital role in protecting cells from oxidative stress and ferroptosis^[48,49]^, we measured GSH and Fe^2+^ levels using colorimetric assays. TAMs showed higher GSH levels and lower Fe^2+^ levels compared to PMs, indicating resistance to ferroptosis. Interestingly, TAMs overexpressing circCCDC7_19-13_ exhibited the opposite trend, with decreased GSH and increased Fe^2+^ levels, making them more susceptible to ferroptosis (Fig. 4F-K). To further confirm the role of ferroptosis, we treated the cells with Ferrostatin-1 (Fer-1), a known ferroptosis inhibitor. The addition of Fer-1 restored the ferroptosis resistance in TAMs overexpressing circCCDC7_19-13_ by reducing lipid peroxidation and Fe^2+^ levels (Fig. 4J-L, S2G-M, available at http://links.lww.com/JS9/E497).

In conclusion, our findings suggest that TAMs have a higher resistance to ferroptosis than PMs. However, circCCDC7_19-13_ overexpression reverses this resistance, making TAMs more prone to ferroptosis. This effect can be rescued by Fer-1, further validating the involvement of ferroptosis in TAM polarization and highlighting circCCDC7_19-13_’s potential role in regulating ferroptosis sensitivity.

**Figure 5.**
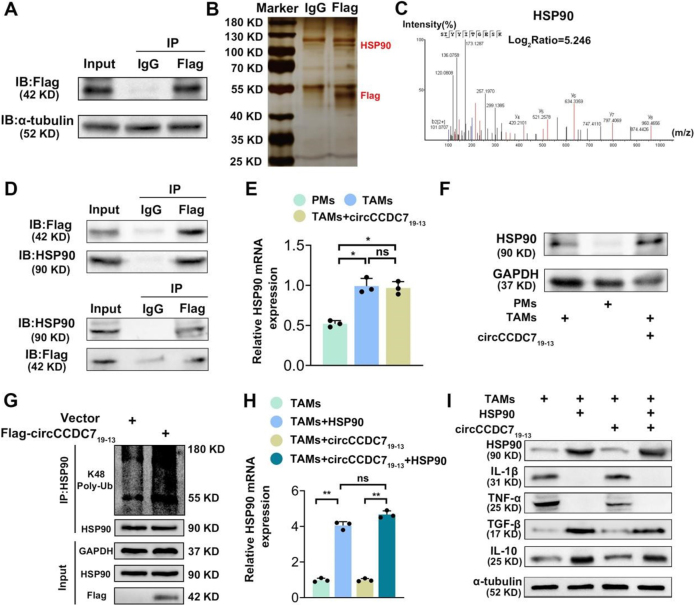
HSP90 is a downstream target of circCCDC7_19-13_ and rescues the effects of circCCDC7_19-13_ on TAM polarization. (A, B) Co-immunoprecipitation (Co-IP) of Flag-tagged circCCDC7_19-13_ and HSP90 in PC3M cells, confirmed by silver staining. HSP90 and Flag-circCCDC7_19-13_ are indicated by red labels. (C, D) Mass spectrometry analysis and Co-IP validate the interaction between endogenous HSP90 and circCCDC7_19-13_. (E) HSP90 mRNA expression levels were measured by RT-qPCR after circCCDC7_19-13_ overexpression in TAMs. (F) Western blot showing the effect of circCCDC7_19-13_ on HSP90 protein levels. (G) Polyubiquitination status of HSP90 was assessed by immunoprecipitation and Western blot analysis. (H) RT-qPCR quantification of HSP90 mRNA levels in TAMs with circCCDC7_19-13_ overexpression and HSP90 rescue. (I) Western blot analysis of M1 markers (IL-1β, TNF-α) and M2 markers (TGF-β, IL-10) after circCCDC7_19-13_ overexpression and HSP90 rescue.

We performed co-immunoprecipitation (Co-IP), silver staining, and mass spectrometry (MS) analysis to identify proteins interacting with circCCDC7_19-13_. HSP90 was identified as a direct binding partner and downstream target of circCCDC7_19-13_, reversing the inhibitory effects of circCCDC7_19-13_ on TAM M2 polarization (Fig. 5A-D). HSP90 is a molecular chaperone involved in protein folding, stabilization, and degradation, maintaining cellular homeostasis under stress conditions^[50]^. Recent studies have linked HSP90 with ferroptosis through its regulation of SLC7A11 and iron metabolism, affecting lipid peroxidation and glutathione balance^[51,52]^. We next explored the relationship between circCCDC7_19-13_ and HSP90. RT-qPCR and Western blot analyses showed that HSP90 expression was elevated in TAMs compared to PMs, while HSP90 levels decreased following circCCDC7_19-13_ overexpression. Conversely, HSP90 rescue experiments restored HSP90 expression levels in circCCDC7_19-13_-overexpressing TAMs (Fig. 5E-F).

To further investigate how circCCDC7_19-13_ regulates HSP90, we evaluated the ubiquitination status of HSP90. Polyubiquitination of HSP90 was significantly enhanced in TAMs overexpressing circCCDC7_19-13_, indicating that circCCDC7_19-13_ promotes proteasomal degradation of HSP90 through K48-linked ubiquitination (Fig. 5G). This suggests that circCCDC719-13 destabilizes HSP90, reducing its stability and altering macrophage polarization. Moreover, recent studies have suggested that HSP90 may play a role in regulating macrophage polarization. Specifically, HSP90 has been shown to interact with and stabilize several proteins that are involved in macrophage polarization, including STAT1, STAT3, and NF-κB, which are key transcription factors involved in the M1/M2 polarization process^[51,52]^. To confirm the impact of circCCDC7_19-13_ on macrophage polarization, we analyzed the cytokine secretion profiles of TAMs. TAMs overexpressing circCCDC7_19-13_ exhibited increased secretion of M1 cytokines (IL-1β, TNF-α) and reduced release of M2 cytokines (TGF-β, IL-10). However, in TAMs co-expressing circCCDC7_19-13_ and HSP90, the elevated M1 cytokine levels were attenuated, and M2 cytokine release was restored, suggesting that HSP90 counteracts the effects of circCCDC7_19-13_ on TAM polarization (Fig. 5H-I).

**Figure 6.**
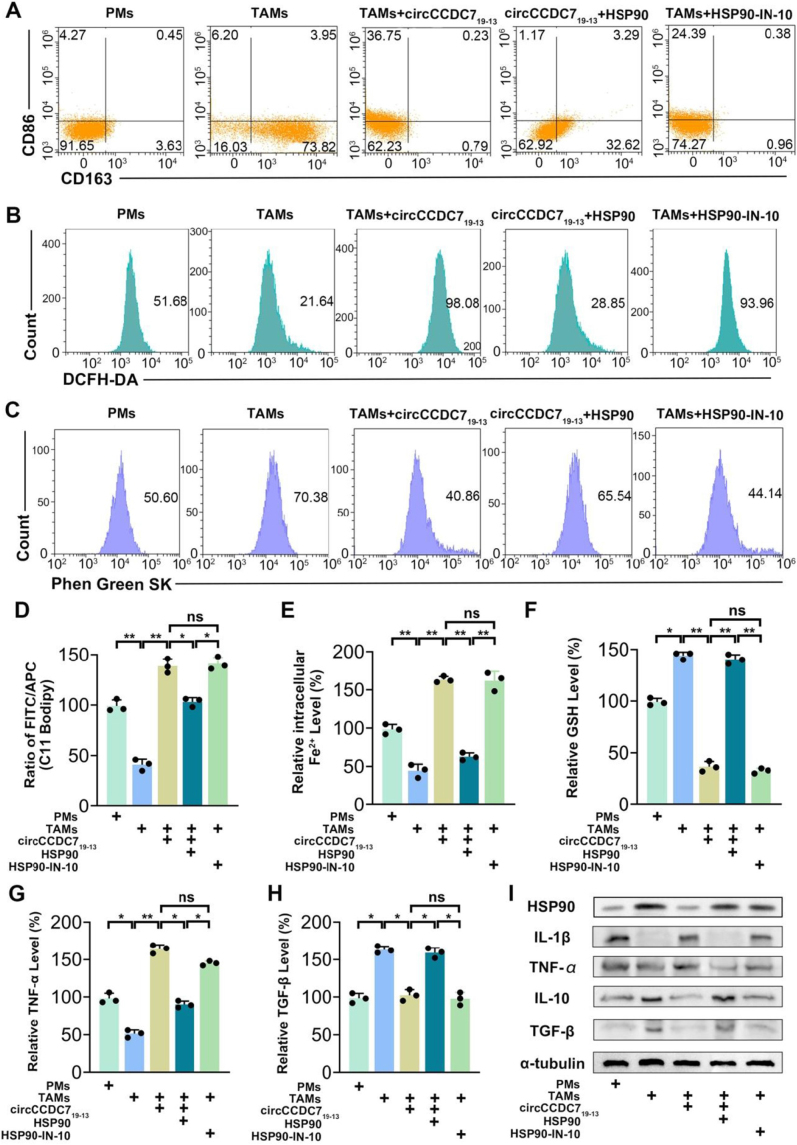
Suppression HSP90 reverses circCCDC7_19-13_ overexpression-induced ferroptosis and polarization of TAMs. (A) Flow cytometry analysis showing the expression of CD86 (M1 marker) and CD163 (M2 marker) across different treatment groups. (B) ROS levels were measured using DCFH-DA staining and analyzed by flow cytometry. (C) Intracellular Fe^2+^ levels were detected using the Phen Green SK assay by flow cytometry. (D) Lipid peroxidation was assessed using the C11 BODIPY assay and presented as the FITC/APC ratio. (E, F) Glutathione (GSH) and Fe^2+^ levels were quantified using colorimetric methods. (G,H) ELISA-based assays measured the secretion levels of TNF-α (M1 cytokine) and TGF-β (M2 cytokine). (I) Western blot confirming the expression levels of HSP90 and cytokines across experimental groups.

Our findings indicate that circCCDC7_19-13_ influences TAM polarization by inducing ferroptosis. To explore the role of HSP90 in this process, we investigated the impact of HSP90 expression and inhibition on ferroptosis and TAM polarization. The results showed that high HSP90 expression reduced intracellular ROS, Fe^2+^ levels, and lipid peroxidation, while increasing glutathione (GSH) levels, indicating enhanced resistance to ferroptosis (Fig. 6B-F). Flow cytometry analysis revealed a decrease in CD86 (M1 marker) and an increase in CD163 (M2 marker) expression in TAMs with high HSP90 expression, suggesting a shift towards M2 polarization (Fig. 6A). Treatment with the HSP90 inhibitor (HSP90-IN-10) reversed these effects, leading to increased ROS, Fe^2+^, and lipid peroxidation, along with reduced GSH levels. This suggests that HSP90 is involved in mediating the ferroptosis resistance and polarization shift induced by circCCDC7_19-13_.

To further confirm these findings, we measured the secretion of M1-type cytokines (TNF-α, IL-1β) and M2-type cytokines (TGF-β, IL-10) using ELISA-based assays and Western blot analysis. The results showed that HSP90 overexpression increased M2 cytokines and suppressed M1 cytokines, while HSP90-IN-10 treatment reversed this trend, restoring M1 polarization (Fig. 6G-I, S2A-F, available at, http://links.lww.com/JS9/E497).These results demonstrate that HSP90 plays a critical role in mediating the effects of circCCDC7_19-13_ on TAM polarization and ferroptosis. Targeting HSP90 with inhibitors like HSP90-IN-10 offers a potential strategy to modulate TAM polarization and alter the tumor microenvironment.

**Figure 7.**
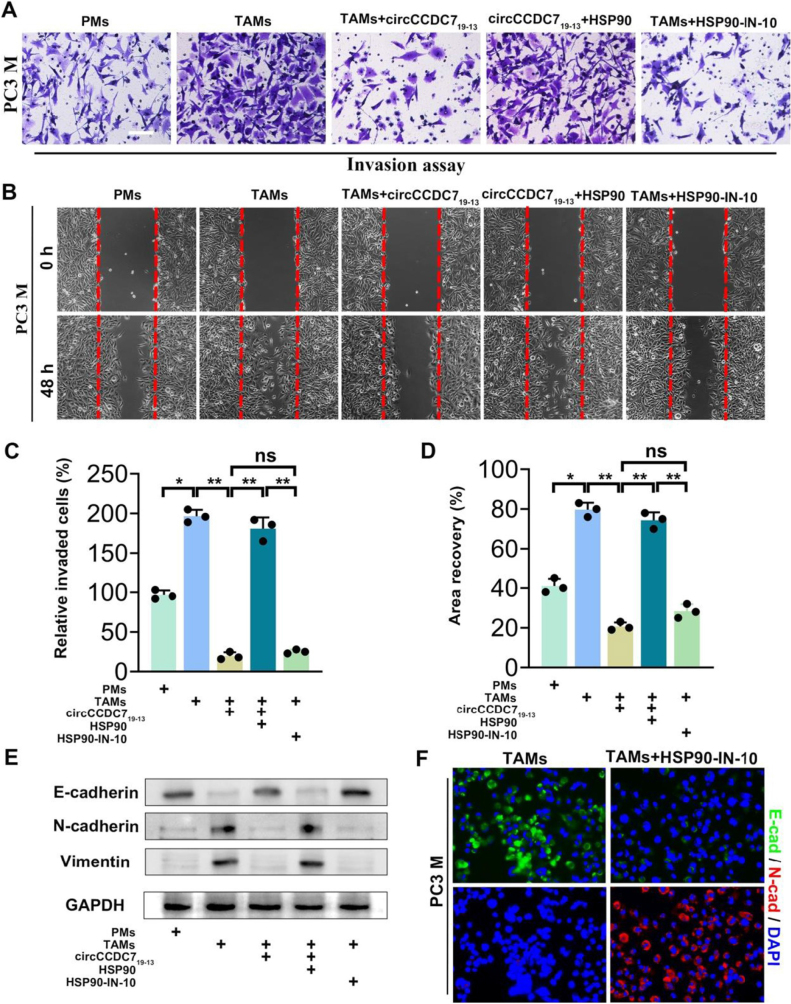
HSP90 reverses the changes in the biological characteristics of prostate cancer cells induced by TAMs with high circCCDC7_19-13_ expression. (A) Transwell invasion assays showing the invasion abilities of PC3M prostate cancer cells co-cultured with different groups of TAMs. (B) Wound healing assays demonstrating the migration abilities of PC3M cells co-cultured with TAMs from different groups, observed at 0 and 48 hours post-scratch (200x magnification). (C) Quantification of invaded cells from the Transwell assays, presented as mean ± SD from three independent experiments. (D) Quantification of area recovery from the wound healing assays, with data expressed as mean ± SD from three independent experiments. (E) Western blot analysis of EMT markers (E-cadherin, N-cadherin, vimentin) in PC3M cells co-cultured with TAMs under the indicated conditions. GAPDH was used as the internal control. (F) Immunofluorescence staining of EMT markers (E-cadherin in green, N-cadherin in red) in PC3M cells co-cultured with TAMs or TAMs treated with HSP90-IN-10, with DAPI staining the nuclei (200x magnification).

After identifying HSP90 as the target of circCCDC7_19-13_, we explored the role of HSP90 in modulating the tumor-promoting abilities of TAMs. Our experiments revealed that HSP90 overexpression enhanced the ability of TAMs to promote invasion and migration of PC3M prostate cancer cells. However, when both circCCDC7_19-13_ and HSP90 were overexpressed in TAMs, the invasion and migration abilities of PC3M cells were reduced to baseline levels (Fig. 7A-D). To further investigate how these changes relate to the epithelial-mesenchymal transition (EMT), we analyzed the expression of EMT markers in PC3M cells. Western blot and immunofluorescence analyses showed that PC3M cells co-cultured with TAMs overexpressing HSP90 exhibited decreased E-cadherin (epithelial marker) expression and increased N-cadherin and vimentin (mesenchymal markers) expression (Fig. 7E,F). Notably, treatment with HSP90-IN-10 partially reversed these EMT changes, restoring E-cadherin levels and reducing N-cadherin expression.

These findings suggest that HSP90 promotes the EMT process in prostate cancer cells by modulating TAM phenotype. Furthermore, HSP90-IN-10 reverses the effects of HSP90, highlighting the role of circCCDC7_19-13_ and HSP90 in controlling EMT and tumor progression. Collectively, our results demonstrate that HSP90 counteracts the effects of circCCDC7_19-13_ on TAM phenotype, enabling TAMs to enhance EMT and promote prostate cancer progression.

To explore the in vivo role of circCCDC7_19-13_ in regulating prostate cancer growth and metastasis via TAM polarization, we performed experiments using tissue-derived macrophages transfected with circCCDC7_19-13_-overexpressing lentivirus. These TAMs were mixed with PC3M prostate cancer cells at a 4:1 ratio and injected into the footpads of mice to establish a popliteal lymph node metastasis model. Tumor growth was monitored every three days, and after 24 days, we harvested the footpad tumors and popliteal lymph nodes (LNs) for histological analysis, including H&E staining and immunohistochemistry (IHC).

**Figure 8.**
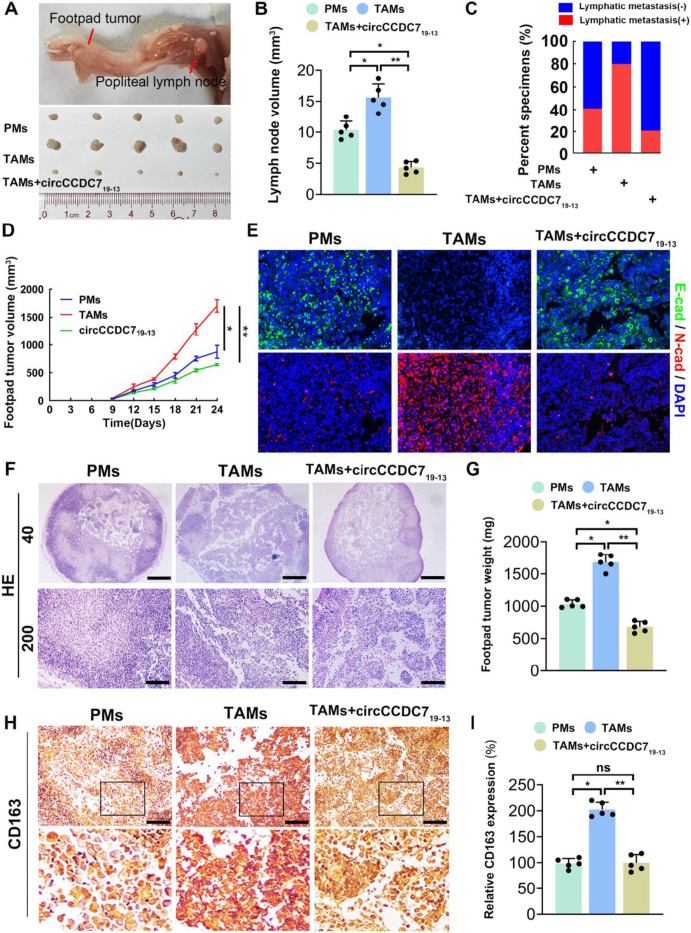
circCCDC7_19-13_ inhibits PC3M prostate cancer cell metastasis by inhibiting M2 macrophage polarization in vivo. (A) Representative images of footpad tumors and popliteal lymph nodes from different groups. (B) Histogram analysis of popliteal lymph node volumes (*n* = 5 per group). Data are presented as mean ± SD. (C) Percentage of lymphatic metastasis in each group (*n* = 5 per group). (D) Tumor growth curves of footpad tumors in different groups (PMs, TAMs, TAMs + circCCDC7_19-13_) measured every 3 days. (E) Representative immunofluorescence images showing E-cadherin (green) and N-cadherin (red) expression in footpad tumors. Nuclei are stained with DAPI (blue). (F) Representative H&E staining images of footpad tumors at 40x and 200x magnifications. (G) Histogram analysis of footpad tumor weights (*n* = 5 per group). (H) Representative immunohistochemical staining images of CD163 in footpad tumors from different groups. (I) Quantification of CD163 expression in footpad tumors from different groups.

Our findings showed that tumors from the TAM group exhibited larger volumes, faster growth, and higher metastatic potential compared to tumors co-cultured with PMs. However, the group with circCCDC7_19-13_-overexpressing TAMs displayed significantly smaller tumors, slower growth rates, and a lower probability of lymph node metastasis (Fig. 8A-D). Furthermore, immunofluorescence staining of EMT markers in tumor tissues indicated that tumors co-cultured with TAMs showed reduced E-cadherin expression and increased N-cadherin expression, which are hallmarks of EMT (Fig. 8E). In contrast, tumors co-cultured with circCCDC7_19-13_-expressing TAMs displayed higher E-cadherin levels and lower N-cadherin expression, further supporting the suppression of EMT and tumor progression.

To determine whether the suppression of prostate cancer growth was related to a shift in TAM polarization, we performed IHC staining for CD163, a marker of M2 macrophages. The results revealed a significant decrease in CD163-positive M2 macrophages in the circCCDC7_19-13_ group, suggesting a reduction in M2-type polarization (Fig. 8H,I). Together, these findings demonstrate that circCCDC7_19-13_ suppresses prostate cancer growth and metastasis by inhibiting the M2 polarization of TAMs, thereby reducing their tumor-promoting effects. This suggests that targeting TAM polarization through circCCDC7_19-13_ could be an effective strategy for controlling prostate cancer progression.

**Figure 9.**
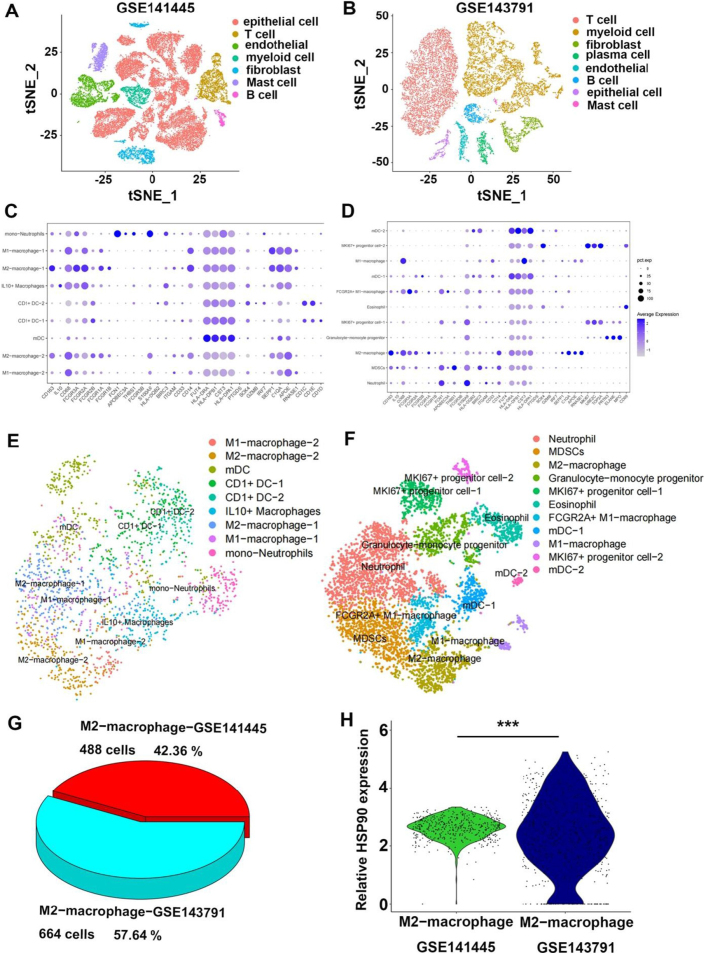
External single-cell sequencing data showed that the expression levels of HSP90 in TAMs of metastatic lesions were significantly higher than those in TAMs of primary prostate cancer. (A,B) t-distributed stochastic neighbor embedding (t-SNE) plots of immune cells from two prostate cancer datasets: primary prostate cancer (GSE141445) and bone metastases from prostate cancer (GSE143791). Cells are color-coded based on their cell types. (C,D) Dot plots showing the expression of marker genes used to define cell populations in the datasets GSE141445 and GSE143791. (E,F) t-SNE plots of myeloid cells from each group, color-coded based on their associated clusters. (G) Pie chart showing the proportion of M2-macrophages in the primary prostate cancer dataset (GSE141445) and bone metastases dataset (GSE143791). (H) Violin plot comparing the expression levels of HSP90 in M2-macrophages between primary prostate cancer (GSE141445) and bone metastases (GSE143791). Data are presented as mean ± SD. ***P < 0.001.

We further validated our findings with external datasets by analyzing the results of single-cell sequencing in public databases (GEO: GSE141445/GSE143791). HSP 90 expression in TAMs was significantly higher in prostate bone metastases than in primary prostate cancer tissues (Fig. 9).

## Discussion

Prostate cancer (PCA) is a leading cause of cancer-related death among men^[53]^, and despite advances in treatment, metastatic PCA remains largely incurable^[54]^. Understanding the mechanisms underlying PCA metastasis is crucial for the development of new drugs and better cancer treatments. The tumor microenvironment (TME) plays a critical role in PCA metastasis, and macrophages are immune cells that can induce cancer cell proliferation and dissemination through infiltration. Macrophages play a “dual role” in cancer development, and the transformation from the M1 to the M2 phenotype shifts their function from inhibiting cancer to promoting cancer. Therefore, blocking this process is considered a potential strategy for cancer treatment^[13,55]^. In prostate cancer, tumor-associated macrophages (TAMs) have been found to contribute to the division and proliferation of tumor cells, leading to the formation of metastatic lesions. Recent studies have shown that adenocarcinoma cells and macrophages work together through cytokine signaling, extracellular matrix remodeling, and immune evasion mechanisms^[56-58]^. Targeting these interactions could be a potential strategy for controlling cancer, which suggests future research directions. However, the molecular mechanisms underlying the transformation of TAMs in the complex TME are still not fully understood.

Tumor-associated macrophages (TAMs) are a crucial component of the tumor microenvironment and have been shown to undergo ferroptosis in response to various stimulation, such as ROS or lipid peroxidation^[59-61]^. This phenomenon has been associated with the regulation of inflammatory responses and clearance of dead cells. Studies have shown that macrophages undergoing ferroptosis secrete IL-1β and IL-18^[62]^, which can stimulate immune responses and tissue repair. Ferroptosis in macrophages is particularly relevant in diseases where macrophages play a crucial role, such as atherosclerosis and cancer. In cancer, macrophages (TAMs) are a very special cell type, and they have been found to promote the growth and infiltration of tumors, aggravating disease. Inducing ferroptosis in tumor-associated macrophages (TAMs) has emerged as a promising therapeutic approach^[47,61,63]^. Nevertheless, the precise mechanisms involved in the induction of ferroptosis in macrophages remain incompletely understood.

Circular RNAs play important roles in regulating gene expression in immune cells, including macrophages, via the post-transcriptional regulation of target genes^[64-67]^. Previous studies have shown that circCCDC7_19-13_ acts as a tumor suppressor in PCA, with in vivo experiments revealing a greater tumor suppressive effect than in vitro experiments. This suggests that circCCDC7_19-13_ has the potential to regulate components of the immune microenvironment and impact antitumor immune responses. Our research showed that TAMs have reduced expression of circCCDC7_19-13_,which alters the polarization state of macrophages. We further investigated the mechanisms and relevant targets of circCCDC7_19-13_ and found that its overexpression increases ROS levels, intracellular Fe^2+^ levels, and lipid peroxidation levels in TAMs, thereby inducing ferroptosis and altering macrophage polarization. We also verified HSP90 as the downstream target of circCCDC7_19-13_, which is involved in regulating macrophage polarization and immune responses. Overexpression of HSP90 in TAMs partially reversed the effects of circCCDC7_19-13_, suggesting that circCCDC7_19-13_ targets HSP90 to induce ferroptosis and M1 polarization. However, the incomplete reversal of these effects by HSP90 indicates that other pathways may also be involved. Compared with existing literature on non-coding RNAs regulating macrophage polarization, our study provides a novel perspective. Most prior studies have focused on miRNAs or linear lncRNAs, such as miR-155, which promotes M1 polarization, or lncRNA MALAT1, which favors M2 polarization in various cancers^[68]^. Only a few studies have explored the roles of circular RNAs in macrophage biology. For instance, circSDHAF2 has been shown to modulate PD-L1 expression in Glioblastoma cancer-associated macrophages, but whether circRNAs directly induce ferroptosis in TAMs was previously unknown^[68]^. Our data therefore expand the functional repertoire of circRNAs by linking circCCDC7_19-13_ to both immune cell reprogramming and ferroptotic regulation, which, to our knowledge, has not been concurrently demonstrated in the context of prostate cancer.^[69-76]^

While our findings provide compelling evidence for the role of circCCDC7_19-13_ in regulating TAM polarization and ferroptosis, we acknowledge certain technical limitations. Attempts to knock down circCCDC7_19-13_ using siRNA in TAMs were hindered by the RNA’s unique circular structure and its low endogenous expression in these cells. These challenges resulted in insufficient knockdown efficiency, preventing us from conducting reliable functional studies using this approach. To overcome this limitation, we are actively exploring CRISPR/Cas13-based methods for circRNA-specific degradation, which offer higher specificity and efficiency. Although this method was not feasible within the timeframe of the current study, we plan to implement it in future research to further validate the functional role of circCCDC7_19-13_ and its downstream targets. This limitation and the potential of CRISPR/Cas13 as a more robust alternative are discussed as future directions in this study.

In conclusion, our study demonstrates that circCCDC7_19-13_ plays a critical role in inducing ferroptosis and reversing M2 polarization in TAMs by regulating HSP90. Importantly, circCCDC7_19-13_ also harbors protein-coding potential, which adds a novel layer of functional relevance and opens up new avenues for therapeutic exploitation. By simultaneously modulating tumor-associated macrophage function and the immunosuppressive tumor microenvironment, circCCDC7_19-13_ represents a promising therapeutic target in prostate cancer. Given these findings, therapeutic strategies designed to enhance circCCDC7_19-13_ activity—such as synthetic RNA mimetics, AAV-mediated overexpression, or peptide-based delivery of its encoded product—could offer synergistic benefits when combined with existing prostate cancer treatments. Future studies should explore these translational strategies to unlock the full clinical potential of circCCDC7_19-13_, particularly in patients with advanced or castration-resistant disease.

## Conclusion

In conclusion, our study uncovers a significant association between circCCDC7_19-13_ expression in tumor-associated macrophages (TAMs) and prostate cancer metastasis. Furthermore, we identify a novel mechanism by which circCCDC7_19-13_ regulates TAM polarization through the induction of ferroptosis via targeting HSP90. Notably, tumor cells suppress circCCDC7_19-13_ expression in TAMs, thereby relieving the inhibitory effect on downstream HSP90. Consequently, HSP90 promotes the M2 polarization of TAMs by conferring resistance to ferroptosis. Subsequently, the secretion of TGF-β and IL-10 by M2-type TAMs facilitates epithelial-mesenchymal transition (EMT) and distant metastasis of prostate cancer cells. These findings provide valuable insights into the plasticity of the tumor microenvironment (TME) and may contribute to the development of effective strategies for the prevention and treatment of metastatic prostate cancer.

## Data Availability

All the data will be provided upon reasonable request.

## Research registration unique identifying number (UIN)

Name of the registry: Effectiveness of Cytoreductive Surgery in Treating Oligometastatic Prostate Cancer: Insights from Quantitive-analysis and Retrospective cohort Studies. Unique Identifying number or registration ID: CRD42023454107. Hyperlink to your specific registration (must be publicly accessible and will be checked): https://www.crd.york.ac.uk/prospero/.

## Patient and public involvement

Patients or the public WERE NOT involved in the design, or conduct, or reporting, or dissemination plans of our research.
